# Transesophageal echocardiography for cardiac herniation occurring during robotic-assisted mitral valve repair: a case report

**DOI:** 10.1186/s40981-023-00594-z

**Published:** 2023-01-13

**Authors:** Kazuto Miyata, Sayaka Shigematsu, Naoki Miyayama

**Affiliations:** Department of Anesthesia, New Heart Watanabe Institute, Hamadayama 3-19-11, Suginami-Ku, Tokyo, Japan

**Keywords:** Cardiac herniation, Transesophageal echocardiography, Double-lumen tube, Robotic-assisted mitral valve repair

## Abstract

**Background:**

Cardiac herniation has been reported in thoracic trauma and after pneumonectomy; however, it is sporadic in cardiac surgery.

**Case presentation:**

A 35-year-old male patient underwent an elective totally endoscopic robotic-assisted mitral valve repair (TERMVR). His hemodynamics were stable after weaning from cardiopulmonary bypass, and no residual mitral valve regurgitation was observed. However, during suturing of the port wound, the patient developed hypotension, which improved with phenylephrine administration. Four-chamber transesophageal echocardiography (TEE) images showed cardiac deformity, and postoperative chest radiography confirmed the dextrocardia. The cardiac herniation was repaired by deflating the left lung and over-inflating the right lung using a double-lumen tube, allowing selective ventilation without re-thoracotomy. The patient was discharged on the sixth postoperative day without complications.

**Conclusions:**

This was a very unusual case of cardiac herniation during TERMVR visualized using distinct TEE images. The cardiac herniation was successfully repaired using a double-lumen tube without re-thoracotomy.

**Supplementary Information:**

The online version contains supplementary material available at 10.1186/s40981-023-00594-z.

## Background

Cardiac herniation has been reported in thoracic trauma [1] and after pneumonectomy [2]. Although cardiac herniation can occur after any minimally invasive cardiac surgery (MICS) involving a pericardial incision, it is sporadic [3–5]. To the best of our knowledge, only four cases have been reported in MICS [3–5].

In our institute, a totally endoscopic robotic-assisted mitral valve repair (TERMVR) is constantly performed; partial removal of the right pericardium post-incision for hemostasis and augmentation of the mitral valve is also standard procedure [6]. Therefore, the pericardium is frequently exposed postoperatively, which could lead to cardiac herniation during the perioperative period. Here, we documented distinct transesophageal echocardiography (TEE) images of the cardiac herniation, which indicated a pronounced left ventricular apex movement. Subsequently, the cardiac herniation was successfully repaired using a double-lumen tube without re-thoracotomy. Written consent was obtained from the patient to publish this case report.

## Case presentation

A 35-year-old male patient (height: 179 cm, weight: 49 kg) with severe mitral regurgitation (MR) was referred to our institution for elective minimally invasive mitral valve repair. He had no relevant medical history. Preoperative laboratory blood test results were all normal. A preoperative TEE revealed severe MR due to a prolapsed anterior leaflet (A3) and a hypoplastic posterior leaflet. Electrocardiography showed normal sinus rhythm with a heart rate (HR) of 75 bpm.

After the induction of general anesthesia, the patient was intubated with a left-sided 37 Fr double-lumen tube to establish one-lung ventilation (OLV). Ventilator settings during OLV included pressure control ventilation (PCV) with positive end-expiratory pressure (PEEP) set to 0 cmH_2_O and an inspiratory to expiratory ratio of 1:2. Percutaneous arterial oxygen saturation (SpO_2_) > 95% was maintained under 100% oxygenation with a maximum airway pressure of 15 cmH_2_O and tidal volume of approximately 300 mL. Immediately after intubation, a TEE probe was inserted, and a central venous catheter and a pulmonary artery catheter were both placed in the left internal jugular vein. Under echocardiographic guidance, a 16 Fr venous cannula was inserted through the right internal jugular vein. Another 16 Fr cannula was inserted into the right femoral artery, and a 27 Fr cannula was inserted into the right femoral vein, which threaded through to the right atrium, guided by TEE. The conventional procedure for cardiopulmonary bypass (CPB) was subsequently performed.

After establishing the CPB, direct aortic clamping was performed using the Chitwood technique. Subsequently, TERMVR via four thoracic ports in the left chest wall using the da Vinci surgical system XI (Intuitive Surgical, Inc., Sunnyvale, CA) was conducted [6]. Cardiac surgeons performed mitral valve leaflet repair through the anterior leaflet neochord reconstruction reinforced with a standard 32-mm MEMO 4D mitral ring (Sorin Biomedica Cardio Srl, Saluggia, Italy) mitral annuloplasty band. After completing the leaflet repairs, the aorta was declamped, autonomous heart activity was re-established, and the patient was weaned from CPB without incident. TEE showed no residual mitral regurgitation (MR).

**Fig. 1:**
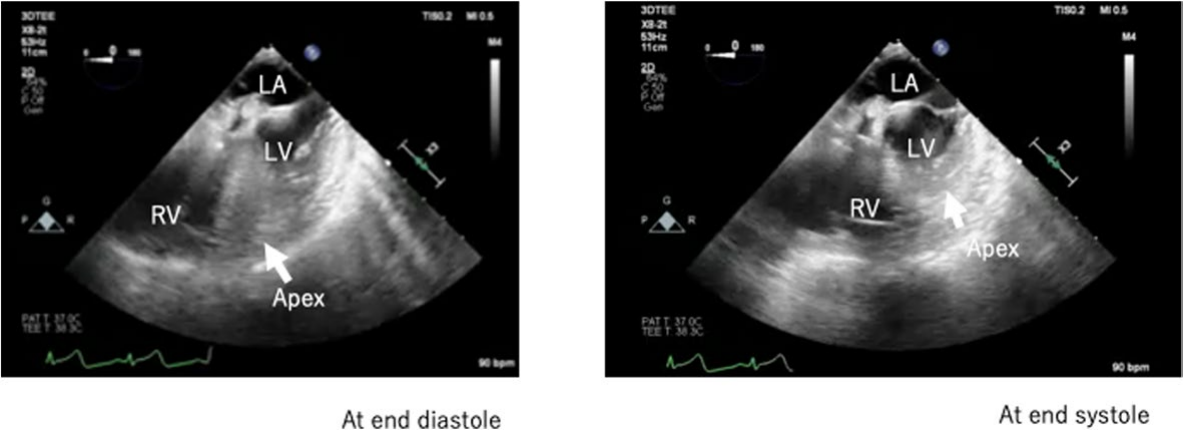
Four-chamber mid-esophageal echocardiographic images at end diastole (left) and end systole (right). The left ventricular apex shifted significantly during the cardiac cycle

**Fig. 2:**
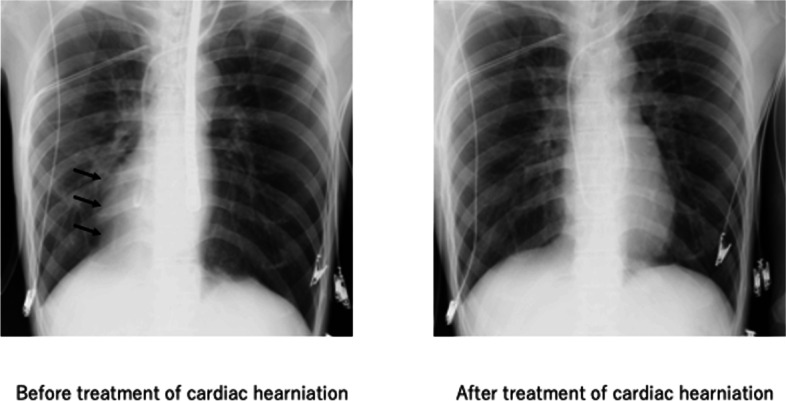
Chest XP before (left) and after (right) treatment of cardiac hernia. The heart in the right hemithorax was repositioned to the normal position after right one-lung ventilation. Black arrow: protrusion of the right cardiac border

Using PCV with a maximum airway pressure of 15 cmH_2_O and PEEP of 0 cmH_2_O during the closure of the thoracic port incisions on the chest wall under OLV to avoid parenchymal lung injury, premature ventricular contractions occurred. The patient’s hemodynamic parameters abruptly decompensated to an arterial, pulmonary artery, and central venous pressures of 46/24 mmHg, 9/1 mmHg, and 10 mmHg, respectively. After suspecting hemorrhage with cardiac tamponade, we immediately verified if the thoracic drain placement was correct and aspirated it at − 20 mmHg. However, no blood was aspirated. TEE was reperformed to determine the cause of the shock. Four-chamber TEE images showed deformity of the left ventricle, distinctly pronounced motion of the left ventricular apex during the cardiac cycle, and absence of pericardial fluid (Fig. 1) (Additional file 1). Transgastric short-axis TEE images showed left ventricular swing; however, wall motion asynergy could not be accurately evaluated (Additional file 2). After frequent phenylephrine dosing (total amount administered, 1 mg), the patient’s hemodynamic parameters gradually stabilized at arterial, pulmonary artery, and central venous pressures of 92/45 mmHg, 20/10 mmHg, and 10 mmHg, respectively. Postoperative chest radiography in the operating room revealed a protrusion of the right cardiac border, confirming a diagnosis of cardiac herniation (Fig. 2).

To correct the cardiac herniation, we attempted to block and deflate the left lung while manually ventilating the right lung with high pressure (> 30 cmH_2_0) using a double-lumen endobronchial tube, which allowed selective ventilation. Consequently, this improved cardiac positioning, as confirmed via TEE, without further wall motion abnormalities noted. Subsequent chest radiography performed in the operating room showed improvement in cardiac herniation (Fig. 2). The patient was extubated on postoperative day 0 and discharged on the sixth postoperative day without any complications.

## Discussion

Here, we highlight two important clinical findings. First, cardiac herniation was visualized using TEE images showing the pronounced movement of the left ventricular apex during the cardiac cycle in the right mini-thoracotomy MICS, which included robotic-assisted mitral valve surgery. Second, the cardiac herniation was effectively repaired by deflating the left lung and hyperinflating the right lung separately using a double-lumen tube without re-thoracotomy.

However, only a few previous studies have reported the rare complications of cardiac herniation after right mini-thoracotomy MICS [3–5]. In this technique, the right side of the pericardium is always incised for cardiac exposure. In our TERMVR procedures, the pericardium is not sutured closed because the autologous pericardium is harvested intraoperatively for suture reinforcement and hemostasis in all cases, and in a few cases, for mitral valve augmentation. At our institute, 796 cases of robotic-assisted mitral valve repairs were performed between May 2014 and May 2022. We did not experience any complications after keeping the right pericardial open [7]. This procedure is standard practice in our institute and was the first case where cardiac herniation occurred through a right pericardial opening at our institute.

The cause of cardiac herniation in our case was unknown. Since we failed to directly observe cardiac herniation through endoscopy, perhaps cardiac herniation occurred because of the left lung over-expansion before the two-lung ventilation was restarted. However, we always used PCV settings during OLV. Here, maximal airway pressure did not exceed 15 cmH_2_O during OLV, and we presume that this airway pressure did not induce overinflation of the left lung.

Previous reports on cardiac herniation in MICS include only one case of hemodynamic deterioration [3–5]. Here, ventricular extrasystoles and hypotension were also recognized. The hypotension after weaning from CPB may have been caused by hemorrhage, cardiac tamponade, or sudden cardiac dysfunction. TEE is a useful tool for diagnosing hemodynamic deterioration during cardiac surgery. However, the typical TEE findings of cardiac herniation are unknown. The pathophysiology of cardiac herniation is a deviation of the left ventricular apex toward the right thoracic cavity.

It has been reported that TEE cannot acquire standard mid-esophageal views and cannot visualize the heart through a transgastric view [5]. Here, the TEE findings on a four-chamber mid-esophageal view showed pronounced left ventricular apex movement, which is stationary in normal conditions, during the cardiac cycle (Fig. 1). The TEE findings on a transgastric short-axis view also showed a swing of the left ventricle caused by left ventricular apical deviation, as observed in acute cardiac tamponade. Furthermore, the TEE images captured in our case are the first to be reported, which could contribute to future diagnosis of cardiac herniation in cases showing pronounced left ventricular apical motion and left ventricular swing.

It has been reported that in patients with trauma, emergency open surgery to repair the cardiac deviation and close the pericardium is the treatment for cardiac herniation [1]. In cardiac herniation after pneumonectomy, the resected pericardium is repaired with an expanded polytetrafluoroethylene sheet [2]. Here, cardiac herniation repair was initiated using a double-lumen tube, routinely used in MICS, to deflate and hyperinflate the left and right lungs, respectively. MICS is frequently performed using a double-lumen endobronchial tube for general anesthesia to facilitate lung isolation. We speculate that although the pericardium is exposed in MICS, no space is created as in pneumonectomy; therefore, cardiac herniation does not recur despite an open pericardium. Therefore, cardiac herniation after mini-thoracotomy MICS can be effectively repaired without re-thoracotomy.

This was a very unusual case of cardiac herniation during TERMVR, visualized using TEE images. Moreover, the cardiac herniation was successfully repaired using a double-lumen tube without re-thoracotomy.

## Supplementary Information

**Additional file 1.** Four-chamber view in transesophageal echocardiography indicates pronounced left ventricular apical motion. LV: left ventricle, LA: left atrium, RV: right ventricle. White arrow: left ventricular apex.

**Additional file 2.** Transgastric short-axis view in transesophageal echocardiography indicates left ventricular swing.

## Data Availability

Data sharing is not applicable to this article as no datasets were generated or analyzed during the current study.

## Abbreviations

TERMVR: Totally endoscopic robotic-assisted mitral valve repair
TEE: Transesophageal echocardiography
MR: Mitral regurgitation
HR: Heart rate
OLV: One-lung ventilation
PCV: Pressure control ventilation
PEEP: Positive end-expiratory pressure
CPB: Cardiopulmonary bypass
MICS: Minimally invasive cardiac surgery
